# Supplementary material for: Quorum sensing integrates environmental cues, cell density and cell history to control bacterial competence
Source: Nat Commun. 2017 Oct 11;8:854. doi: 10.1038/s41467-017-00903-y (PMC5636887; doi:10.1038/s41467-017-00903-y)
Supplement: Supplementary file 1 — Supplementary Information [file 41467_2017_903_MOESM1_ESM.pdf]

Supplementary Note 1

Model description

Our model consists of two components. At the cellular level, the model keeps track of the intracellular number of proteins involved in competence regulation. At the population-level, it keeps track of the population density and the extracellular CSP concentration. It extends the only previous model in pneumococcal competence[1] by explicitly including population growth and by modelling the interactions between ComE and its phosphorylated form with the gene promoters of the *comAB*, *comCDE* and *ComX* operons. See Supplementary Tables 2 and 3 for all the parameter values used.

A. Cell-level component

We model the signal transduction network that leads from CSP detection to the increased expression of the *comAB*, *comCDE* and *ComX* operons (Figure 1). Importantly, we model the dynamics of the interaction between ComE and phosphorylated ComE (ComE∼P) with the promoters of these operons (Supplementary Figure 11). We assume that the highest rate of transcription for any of these promoters occurs when they are bound to the phosphorylated ComE dimer. Otherwise, we assume that any other configuration of the gene promoter results in low transcription rate. We assume that all the promoter configurations are in quasi-steady state because the number of proteins is much higher than the number of promoters. Also, we assume that the mRNA concentration is in quasi-steady state compared to the protein concentrations for all the genes because the turnover rate of mRNA in the cell is high.

We will use the following general notation,

Parameter	Description
β_x^0	Basal transcription rate of mRNA coding for protein x (when promoter is not bound to the ComE∼P dimer)
β_x	Transcription rate of mRNA coding for protein x when the promoter binds the ComE∼P dimer
Y_{EP}^x	Fraction of total gene promoter x bound to the ComE∼P dimer
g_x	Number of copies of promoter x per cell
σ_x	Translation rate of mRNA coding for protein x
δ_x^R	Degradation rate of mRNA coding for protein x
δ_x	Degradation rate of protein or complex x

<u>CSP detection and phosphorylation of ComE</u>

Synthesis of ComD

ComD is synthesized from the promoter of *comCDE*. The membrane receptor of CSP is formed by two units of ComD and is phosphorylated by the binding to CSP.

$$\frac{dComD}{dt} = \frac{\sigma_D\, g_{CDE}}{\delta_D^R}(\beta_D^0(1 - Y_{EP}^{CDE}) + \beta_D Y_{EP}^{CDE}) - 2d_D ComD^2 + 2d_D^- ComD^{dim} - \delta_D ComD$$

$$\frac{dComD^{dim}}{dt} = d_D\, ComD^2 - d_D^- ComD^{dim} - \kappa\, CSP\, ComD^{dim} + \lambda\, ComDP^{dim} ComE - \delta_{Ddim} ComD^{dim}$$

$$\frac{dComDP^{dim}}{dt} = \kappa\, CSP\, ComD^{dim} - \lambda\, ComDP^{dim} ComE - \delta_{DPdim} ComDP^{dim}$$

where $ComD^{dim}$ and $ComDP^{dim}$ stand for the number of proteins of the ComD dimer and its phosphorylated form respectively. Rate constants d_D and d_D^- correspond to the rates of dimerization and disassociation of the

ComD dimer, λ is the rate of phosphorylation of ComE by ComDP^{dim} and κ is the rate of binding of CSP to ComD^{dim}.

Synthesis of ComE and transfer of phosphate group to ComE

ComE is synthesized from the promoter of *comCDE*. As a monomer, ComE is phosphorylated by ComD. Then it dimerises, and the dimer upregulates the transcription of different gene promoters.

$$\frac{dComE}{dt} = \frac{\sigma_E\, g_{CDE}}{\delta^R_E}(\beta^0_E(1 - Y^{CDE}_{EP}) + \beta_E Y^{CDE}_{EP}) - \lambda\, ComDP^{dim} ComE - \delta_E ComE$$
$$\frac{dComEP}{dt} = \lambda\, ComDP^{dim} ComE - 2d_{EP}\, ComEP^2 + 2d^-_{EP} ComEP^{dim} - \delta_{EP} ComEP$$
$$\frac{dComEP^{dim}}{dt} = d_{EP}\, ComEP^2 - d^-_{EP} ComEP^{dim} - \delta_{EPdim} ComEP^{dim}$$

where *ComEP* and *ComEP*^{dim} stand for the number of proteins of ComE$\sim$P and its dimer form respectively. d_{EP} and d^-_{EP} are the rates of dimerization and disassociation of the ComE$\sim$P dimer respectively. It is assumed that the number of ComE and ComE$\sim$P molecules is not reduced due to binding to the promoters.

CSP production and export

Synthesis of ComAB

Both ComA and ComB are synthesised from the promoter of *comAB*. They bind to form the membrane protein ComAB that modifies ComC and exports CSP from the cell.

$$\frac{dComA}{dt} = \frac{\sigma_A\, g_{AB}}{\delta^R_A}(\beta^0_A(1 - Y^{AB}_{EP}) + \beta_A Y^{AB}_{EP}) - d_{AB} ComA\, ComB + d^-_{AB} ComAB - \delta_A ComA$$
$$\frac{dComB}{dt} = \frac{\sigma_B\, g_{AB}}{\delta^R_B}(\beta^0_B(1 - Y^{AB}_{EP}) + \beta_B Y^{AB}_{EP}) - d_{AB} ComA\, ComB + d^-_{AB} ComAB - \delta_B ComB$$
$$\frac{dComAB}{dt} = d_{AB} ComA\, ComB - d^-_{AB} ComAB - \delta_{AB} ComAB$$

where d_{AB} is the rate at which ComA and ComB form the ComAB complex and d^-_{AB} is the rate at which the complex disassociates.

Synthesis of ComC and export of CSP

ComC is synthesized from the promoter of *comCDE* and is modified and exported to the extracellular space by ComAB. We assume that the reaction for CSP export is:

$$ComAB + ComC \underset{k_e^-}{\overset{k_e^+}{\rightleftharpoons}} ComAB\text{-}ComC \overset{r_e}{\rightarrow} ComAB + CSP$$

We further assume that the limiting rate is r_e and that the amount of ComC is much higher than the amount of ComAB. Accordingly, the rate at which CSP is exported from the cell follows Michaelis-Menten kinetics, such that

$$\frac{dComC}{dt} = \frac{\sigma_C\, g_{CDE}}{\delta^R_C}(\beta^0_C(1 - Y^{CDE}_{EP}) + \beta_C Y^{CDE}_{EP}) - \frac{r_e\, ComAB\, ComC}{ComC + k_e} - \delta_C ComC$$

where $k_e = \frac{k_e^- + r_e}{k_e^+}$.

Synthesis of ComX

ComX is synthesized from the promoter of *comX*. ComX directs the synthesis of late competence genes some of which are required for uptake of exogenous DNA.

$$\frac{dComX}{dt} = \frac{\sigma_X\, g_X}{\delta_X^R}(\beta_X^0(1 - Y_{EP}^X) + \beta_X Y_{EP}^X) - \delta_X ComX$$

Interactions of ComE and ComE∼P with the gene promoters

The gene promoter of *comCDE* which we will refer as P_c has two high-affinity sites for binding of both ComE and ComE∼P, while the gene promoter of *comX*, P_x, has one high-affinity site and one low-affinity site[2]. ComE∼P binds as a dimer to both gene promoters while ComE binds as a monomer (Supplementary Fig. 11).

Promoter with two high affinity sites: P_c

Variables:
P_c^0: Free *comCDE* promoter
P_c^{EP}: *comCDE* promoter bound to the ComE∼P dimer
P_c^E: *comCDE* promoter bound to one molecule of ComE
P_c^{E2}: *comCDE* promoter bound to two molecules of ComE

Parameters:
k_c^{+EP}: Rate of binding of the ComE∼P dimer to P_c
k_c^{-EP}: Rate of unbinding the ComE∼P dimer from P_c
k_h^{+E}: Rate of binding of ComE to one site of P_c (high affinity)
k_h^{-E}: Rate of unbinding of ComE from one site of P_c (high affinity)

We assume that the two units of the ComE∼P dimer bind at the same time to the two sites of the promoter P_c. This is, we assume that the binding of the second unit of the ComE∼P dimer is *fast* compared to the binding of the first unit, which is the limiting rate of the binding process. For ComE we explicitly model the two separate bindings because ComE binds as a monomer to both sites of the gene promoter. We make the same assumption for P_x.

Then, the equations for this promoter are:

$$\frac{dP_c^0}{dt} = -k_c^{+EP} P_c^0 ComEP^{dim} + k_c^{-EP} P_c^{EP} - 2k_h^{+E} P_c^0 ComE + k_h^{-E} P_c^E \quad (1)$$

$$\frac{dP_c^{EP}}{dt} = k_c^{+EP} P_c^0 ComEP^{dim} - k_c^{-EP} P_c^{EP} \quad (2)$$

$$\frac{dP_c^E}{dt} = 2k_h^{+E} P_c^0 ComE - k_h^{-E} P_c^E + 2k_h^{-E} P_c^{E2} - k_h^{+E} P_c^E ComE \quad (3)$$

$$\frac{dP_c^{E2}}{dt} = -2k_h^{-E} P_c^{E2} + k_h^{+E} P_c^E ComE \quad (4)$$

We calculate the fraction of the promoter that is in each of the different configurations when the system reaches

the steady-state. The steady-state values for each configuration are denoted by $\hat{P}_c^0, \hat{P}_c^{EP}, \hat{P}_c^E, \hat{P}_c^{E2}$.

From (4), it follows that $\hat{P}_c^{E2} = \dfrac{k_h^{+E}\hat{P}_c^E ComE}{2k_h^{-E}}$.

Adding (3) and (4) and solving at the equilibrium for $\hat{P}_c^E$ yields $\hat{P}_c^E = \dfrac{2k_h^{+E}\hat{P}_c^0 ComE}{k_h^{-E}}$.

Finally, setting (2) to 0 and solving yields $\hat{P}_c^{EP} = \dfrac{k_c^{+EP}\hat{P}_c^0 ComEP^{dim}}{k_c^{-EP}}$.

We introduce the following dimensionless variables to simplify the previous equations,

$$E_H = \frac{ComE}{K_H^E} \quad \text{and} \quad EP_C = \frac{ComEP^{dim}}{K_C^{EP}}$$

where $K_H^E = \frac{k_h^{-E}}{k_h^{+E}}$ and $K_C^{EP} = \frac{k_c^{-EP}}{k_c^{+EP}}$ are the disassociation constants for the binding of ComE (to a high affinity site) and the ComE∼P dimer to P_c respectively.

Then, the fraction of *comCDE* promoter that is bound to the ComE∼P dimer when the system is in the steady-state, Y_C^{EP}, is,

$$\begin{aligned} Y_C^{EP} &= \frac{P_c^0 EP_C}{P_c^0 EP_C + P_c^0 + 2P_c^0 E_H + P_c^0 {E_H}^2} \\ &= \frac{EP_C}{EP_C + (1+E_H)^2} \end{aligned}$$

Analogously, the fraction of promoter that is free is $Y_C^0 = \dfrac{1}{EP_C + (1+E_H)^2}$,

and the fraction of promoter that is bound to at least one ComE molecule is $Y_C^E = 1 - Y_C^0 - Y_C^{EP}$.

Promoter with one high affinity and one low affinity site: P_x

Variables:
P_x^0: Free *comX* promoter
P_x^{EP}: *comX* promoter bound to the ComE∼P dimer
P_x^{Eh}: *comX* promoter bound to one molecule of ComE at the high affinity site
P_x^{El}: *comX* promoter bound to one molecule of ComE at the low affinity site
P_x^{E2}: *comX* promoter bound to two molecules of ComE

Parameters:
k_x^{+EP}: Rate of binding of the ComE∼P dimer to P_x
k_x^{-EP}: Rate of unbinding of the ComE∼P dimer from P_x
k_l^{+E}: Rate of binding of ComE to the low affinity site of P_x
k_l^{-E}: Rate of unbinding of ComE from the low affinity site of P_x

We assume that the rates of binding and unbinding of ComE to a site with high affinity are the same for both gene promoters, P_x and P_c. Thus, the rates of binding and unbinding of ComE from the high affinity site of P_x are k_h^{+E} and k_h^{-E} respectively.

The equations for this promoter are:

$$\frac{dP_x^0}{dt} = -k_x^{+EP} P_x^0 ComEP^{dim} + k_x^{-EP} P_x^{EP} - k_h^{+E} P_x^0 ComE - k_l^{+E} P_x^0 ComE + k_h^{-E} P_x^{Eh} + k_l^{-E} P_x^{El}$$
$$\frac{dP_x^{EP}}{dt} = k_x^{+EP} P_x^0 ComEP^{dim} - k_x^{-EP} P_x^{EP}$$
$$\frac{dP_x^{El}}{dt} = k_l^{+E} P_x^0 ComE - k_l^{-E} P_x^{El} + k_h^{-E} P_x^{E2} - k_h^{+E} P_x^{El} ComE$$
$$\frac{dP_x^{Eh}}{dt} = k_h^{+E} P_x^0 ComE - k_h^{-E} P_x^{Eh} + k_l^{-E} P_x^{E2} - k_l^{+E} P_x^{Eh} ComE$$
$$\frac{dP_x^{E2}}{dt} = k_h^{+E} P_x^{El} ComE + k_l^{+E} P_x^{Eh} ComE - (k_h^{-E} + k_l^{-E}) P_x^{E2}$$

We introduce the following dimensionless variables,

$$E_L = \frac{ComE}{K_L^E} \quad \text{and} \quad EP_X = \frac{ComEP^{dim}}{K_X^{EP}}$$

where $K_L^E = \frac{k_l^{-E}}{k_l^{+E}}$ and $K_X^{EP} = \frac{k_x^{-EP}}{k_x^{+EP}}$ are the disassociation constants for the binding of ComE to a low affinity site and the ComE∼P dimer to P_x respectively.

Then, the fraction of *comX* promoter that is bound to the ComE∼P dimer when the system is in steady-state is,

$$Y_X^{EP} = \frac{EP_X}{EP_X + (1 + E_L)(1 + E_H)}$$

The fraction of promoter that is free is $Y_X^0 = \dfrac{1}{EP_X + (1 + E_L)(1 + E_H)}$,
and the fraction of promoter that is bound to at least one ComE molecule is $Y_X^E = 1 - Y_X^0 - Y_X^{EP}$.

ComAB promoter

Less is known about the specific details of the interaction of the promoter of *comAB* with ComE and the ComE∼P dimer. We assume, analogously to P_x, that this promoter has a high affinity and a low affinity binding site for ComE. Therefore, $Y_{AB}^{EP} = Y_X^{EP}$. We make this assumption because the promoter sequence of one of the binding sites contains a pair of mismatched bases relative to the consensus sequence of the ComE binding sites[2]. This would be consistent with the observation that the response of the *comCDE* promoter to CSP is stronger than the one of the *comAB* promoter[2].

B. Population-level component

The population-level component of the model keeps track of the population density and the extracellular concentration of CSP. Since the total volume of cells is low relative to the volume of the medium, we assume that the external volume remains constant.

Population density

Population growth is described by a logistic growth equation

$$\frac{dn}{dt} = r_g n(1 - n)$$

where r_g is the growth rate of the population and n is the population density normalised by the carrying ca-

pacity of the medium. The rate of protein degradation (due to cell division) equals $r_g = \mu \log 2$ (where μ is the doubling rate).

Extracellular concentration of CSP

$$\frac{dCSP}{dt} = \alpha\, n \left(\frac{r_e\, ComAB\, ComC}{ComC + k_e} \right) - \delta_{CSP} CSP$$

α is a scaling factor to convert the total number of CSP molecules produced by the population per second (population density times the total number of CSP molecules produced by a single cell per second) into the change in the CSP concentration per second.

C. Environmental effects on competence

Effect of pH in the model

We inferred that pH mainly affects CSP export rather than detection based on the experiment presented in Supplementary Figure 8 (see also section on pH in the main text). Therefore, we implemented the effect of pH by modifying the rate r_e at which ComC is exported from the cell as CSP. However, note that pH-dependent CSP export and detection are equivalent in the model. This can be seen by considering the alternative model, in which CSP export is pH-dependent. In such a model, where r_e depends on the pH, the variable for *CSP* can be rescaled by introducing a new variable CSP', in the following way:

$$CSP' = \frac{\gamma\, CSP}{r_e(\text{pH})}$$

Then, the model equations for CSP' production and detection can be written as:

$$\frac{dCSP'}{dt} = \alpha\, n\, \gamma \left(\frac{ComAB\, ComC}{ComC + k_e} \right) - \delta_{CSP} CSP'$$

$$\frac{dComD^{dim}}{dt} = d_D\, ComD^2 - d_D^{-} ComD^{dim} - \text{K(pH)}\, CSP'\, ComD^{dim} + \lambda\, ComDP^{dim} ComE - \delta_{Ddim} ComD^{dim}$$

$$\frac{dComDP^{dim}}{dt} = \text{K(pH)}\, CSP'\, ComD^{dim} - \lambda\, ComDP^{dim} ComE - \delta_{DPdim} ComDP^{dim}$$

where $\text{K(pH)} = \frac{\kappa\, r_e(\text{pH})}{\gamma}$. Note that, in the rescaled alternative model, the parameter that depends on the pH is the one for CSP detection. This shows that the alternative model, with pH-dependent CSP export, is mathematically equivalent to our original model, with pH-dependent CSP production, so that both ways of implementing pH-dependency lead to exactly the same biological conclusions.

Effect of antibiotics in the model

HPUra stalls the replication fork, increasing the copy number of *comAB, comCDE* and *comX*, as these genes are near the origin of replication[3]. Slager *et al.*[3] estimate that this increase can be by a factor of 3 or more. Since we are using lower concentrations of HPUra, we assume that in the presence of this antibiotic the promoter copy number of all these genes (g_{AB}, g_{CDE} and g_X) doubles.

The proposed mechanism of action of streptomycin is to decrease the rate of degradation of CSP by HtrA[4]. Hence, in the model we assume that the rate of CSP degradation δ_{CSP} halves in the presence of streptomycin.

Strain/plasmid	Relevant genotype	Reference
D39	Serotype 2 strain	Avery *et al.*[5]
DSM2	Δ*bgaA*::P_{ssbB}-*luc-gfp*	This study
DLA3	Δ*bgaA*::P_{ssbB}-*luc*	Slager *et al.*[3]
ADP243	Δ*comC::ery*	This study
ADP62	Δ*bgaA*::P_{ssbB}-*luc*, Δ*comC::ery*	This study
ADP49	PMEN14 with *ssbB-luc*	This study
ADP51	*S. mitis* with *ssbB-luc*	This study
ADP25	Δ*cps::chl*	This study
ADP26	Δ*bgaA*::P_{ssbB}-*luc*, Δ*cps::chl*	This study
ADP92	PMEN14 with *ssbB-luc*, Δ*cps::chl*	This study
ADP249	P_{ssbB}-*ssbB-gfp*	This study
ADP151	P_{ssbB}-*ssbB-gfp*, Δ*cps::chl*	This study
ADP235	Δ*cep::p3-mkate2*	This study
ADP244	Δ*cep::p3-mkate2*, Δ*cps::chl*	This study
ADP245	Δ*cep::p3-mkate2*, P_{ssbB}-*ssbB-gfp*	This study
ADP246	Δ*cep::p3-mkate2*, Δ*cps::chl*, P_{ssbB}-*ssbB-gfp*	This study
ADP247	Δ*cep::p3-mkate2*, P_{ssbB}-*ssbB-gfp,* Δ*comC::ery*	This study
ADP248	Δ*cep::p3-mkate2,* Δ*cps::chl*, P_{ssbB}-*ssbB-gfp,* Δ*comC::ery*	This study
ADP95	Δ*bgaA*::P_{ssbB}-*luc* , Δ*prsA::lacI-gm*	This study
ADP112	Δ*bgaA*::P_{ssbB}-*luc*, Δ*cep::*P_{lac}-*comC*, Δ*comC::ery*	This study
ADP107	Δ*bgaA*::P_{ssbB}-*luc* , Δ*cep*::P_{lac}-*comCDE*, Δ*comCDE::chl*	This study
MK356	Δ*bgaA*::P_{ssbB}-*luc-gfp*, Δ*htrA::ery*	This study

Supplementary Table 1: Strains used in this study.

Protein	σ_x/δ_x^R	$\beta_x\, g_x$	$\beta_x^0\, g_x$
ComA	10	0.5	0.015
ComB	15	0.5	0.015
ComC	150	1	0.03
ComD	15	1	0.03
ComE	30	1	0.03
ComX	50	0.5	0.015

Supplementary Table 2: Transcription and translation rates for all proteins in the model. σ_x/δ_x^R corresponds to the number of proteins produced per mRNA molecule. Martin *et al.*[2] estimated the amount of ComE and ComD monomers in a competent cell and they report a ComE/ComD ratio of approximately 2. Since ComD and ComE are under the control of the same promoter we assume that the differences in total number arise during protein translation. Less is known about the stochiometry of ComA, ComB, ComC and ComX so we assume that the differences in translation are proportional to the size of the proteins (aa) relative to ComE. $\beta_x\, g_x$ corresponds to the number of mRNA molecules transcribed per second when all the promoters inside the cell are bound to ComE$\sim$P. Given the rate of protein degradation (which we assume to be the same for all the proteins, see Supplementary Table 3) and the number of proteins per mRNA we adjusted the rate of transcription of *comCDE* to match the estimates of ComE and ComD monomers in a competent cell presented by Martin *et al.*[2]. In this study they also estimated a two-fold difference between the transcription rate of *comCDE* and *comX*. We assumed that *comAB* has the same transcription rate as *comX*. Note that these values are within the range of measured transcription rates in other bacteria[6]. $\beta_x^0\, g_x$ corresponds to the number of mRNA molecules transcribed per second when all the promoters inside the cell are free or bound to ComE. Martin *et al.*[2] estimated an approximate 30-fold difference between the number of ComE and ComD monomers in competent and non-competent cells. We use this estimate to set the basal transcription rate β_x^0 from β_x.

Parameter (Symbol)	Value	Source
Degradation rate of protein or complex x (δ_x)	$4.4 \times 10^{-4}\, s^{-1}$	Doubling time of 26 min *
Degradation rate of CSP (δ_{CSP})	$5.5 \times 10^{-4}\, s^{-1}$	Assigned $^\bullet$
Michaelis constant for CSP export (k_e)	100000 #	Assigned
Export rate of CSP after ComC binds ComAB (r_e)	$5 \times 10^{-3}\, s^{-1}$	Assigned
Rate of binding of CSP to the ComD dimer (κ)	$1 \times 10^{-4} [\,]^{-1} s^{-1}$	Assigned
Rate of phosphorylation of ComE by the phosphorylated ComD dimer (λ)	$1 \times 10^{-4} \#^{-1} s^{-1}$	Assigned
Rate of formation of ComAB complex (d_{AB})	$0.6\, \#^{-1} s^{-1}$	Assigned $^\triangle$
Rate of disassociation of ComAB complex (d_{AB}^{-})	$0.1\, s^{-1}$	Assigned $^\triangle$
Rate of formation of ComD dimer (d_D)	$0.6\, \#^{-1} s^{-1}$	Assigned $^\triangle$
Rate of disassociation of ComD dimer (d_D^{-})	$0.1\, s^{-1}$	Assigned $^\triangle$
Rate of formation of ComE∼P dimer (d_{EP})	$0.6\, \#^{-1} s^{-1}$	Assigned $^\triangle$
Rate of disassociation of ComE∼P dimer (d_{EP}^{-})	$0.1\, s^{-1}$	Assigned $^\triangle$
K_d for binding of ComE∼P to P_c (K_c^{EP})	5800 #	Martin *et al.*[2] $^\circ$
K_d for binding of ComE∼P to P_x (K_x^{EP})	53000 #	Martin *et al.*[2] $^\circ$
K_d for binding of ComE to a high affinity binding site (K_H^E)	27000 #	Martin *et al.*[2] $^{\circ,\times}$
K_d for binding of ComE to a low affinity binding site (K_L^E)	138000 #	Martin *et al.*[2] $^{\circ,\times}$
Volume of a cell (V_S)	1×10^{-15} L	Kubitschek and Friske[7]
Growth rate (r_g)	$4.4 \times 10^{-4}\, s^{-1}$	Doubling time of 26 min
Scaling factor (α)	$0.5\ [\,]\, \#^{-1}$	Assigned

Supplementary Table 3: Other parameters values.

* We assume that there is no further protein degradation so this term only corresponds to dilution due to cell division.

$^\bullet$ Average CSP lifetime of 30min.

$^\triangle$ We assume that the dissociation constant K_d for all these bindings is 10nM which is a typical value for dimeric proteins[8]. We translate this number into number of molecules per cell using an estimate for the volume of an *E. coli* cell of 1×10^{-15} L[7]. We further assume that dimerisation occurs fast relative to other processes like phosphorylation or protein degradation so we set the rate of dimer dissociation to 0.1 s^{-1} and calculate the rate of dimer formation using K_d.

$^\circ$ We tried to infer these values in terms of the number of proteins per cell from the K_d values reported by Martin *et al.*[2] (which are in nM) and assuming a cell volume of 1×10^{-15} L. This yields very low numbers relative to the total amount of proteins present in the cell (given the values in Table S2) so we increased the resulting numbers by a factor of 100 while keeping the same ratio.

$^\times$ Since Martin *et al.*[2] only provide an estimate for the the K_d of the binding of ComE to the *comCDE* and *comX* promoters but do not provide specific estimates for the two binding sites, we assume that the K_d for the *comCDE* promoter corresponds to K_H^E and calculate K_L^E assuming that the K_d of the *comX* promoter corresponds to the average between K_H^E and K_L^E.

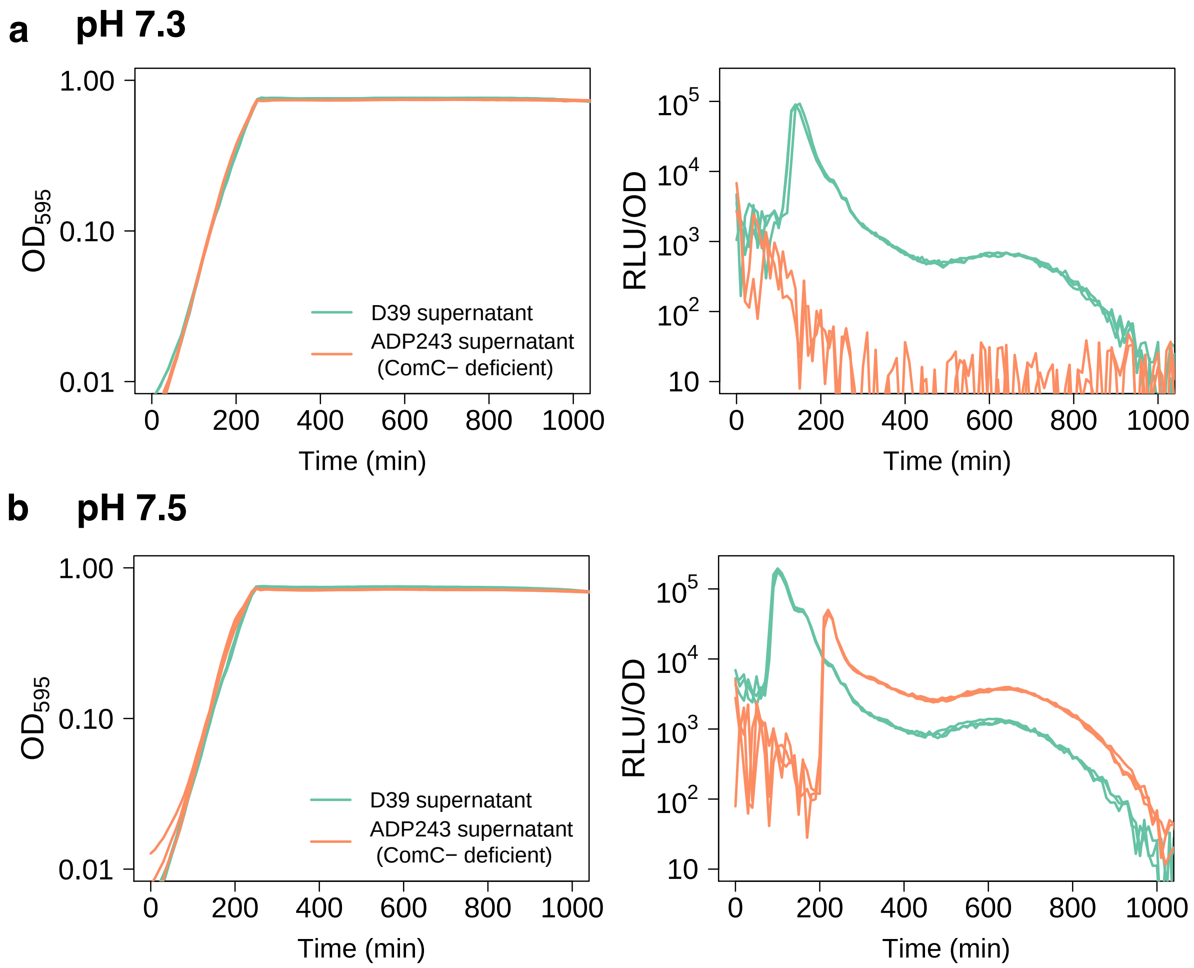

Supplementary Figure 1: D39 secretes CSP to the extracellular space. Growth curves and competence expression measured as relative luminescence units normalised by cell density (RLU/OD) for DSM2 cells growing in 2x concentrated C+Y mixed at a 1:1 ratio with cell-free supernatant from two cultures: one where competence developed, D39 strain grown at pH 7.9, and one where competence did not develop, *comC*-deficient D39 (ADP243) grown at pH 7.9. Three replicates are shown per condition. In a) the initial pH of the medium is adjusted to 7.3. Competence does not develop naturally at this pH (see pH section in the text) but it developed when supernatant from the competent D39 culture was added to the medium. In b) the initial pH of the medium is adjusted to 7.5. Although at this pH competence develops both with the supernatant of the competent and non-competent cultures, it develops faster in the first case. This suggests that the supernatant from the competent D39 culture contained CSP that lead to competence induction of DSM2 at pH 7.3 and sped up competence induction at pH 7.5.

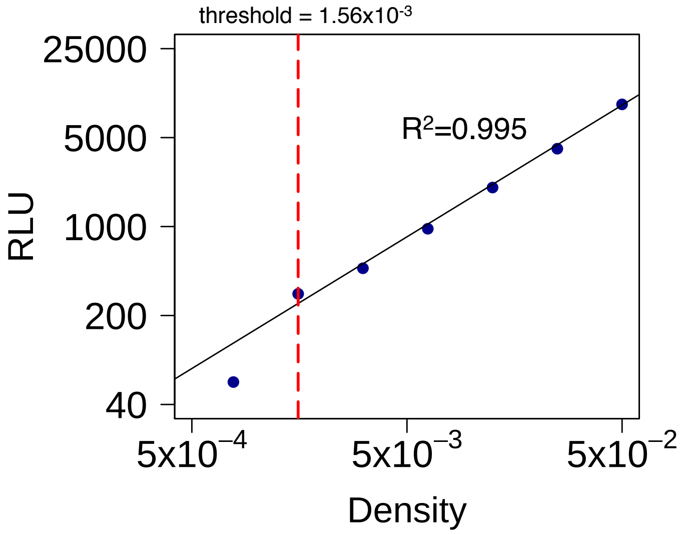

Supplementary Figure 2: Sensitivity of the Tecan Infinite 200 PRO luminometer. RLU signal of DSM2 cells precultured in acid C+Y (pH 6.8), incubated with CSP for 10 minutes and inoculated at different densities in the plate reader. RLU is directly proportional to the cell density up to a density of OD_{595} 1.56×10^{-3}. Below this threshold the RLU signal is lower than expected from the proportionality between cell density and RLU indicating that the luminometer cannot detect all light produced by the population. We performed the same experiment by diluting competent cells in medium with non-competent DSM2 cells (instead of cell-free medium). The total cell density (adding competent and non-competent cells) was fixed to 10^{-1} for all dilutions. The difference in RLU with respect to the data for competent cells diluted in cell-free medium was less than 8% for all dilutions. This indicates that even if the light would come from a subpopulation of competent cells our luminometer would be able to detect it as long as the size of this subpopulation is above the density threshold for detection.

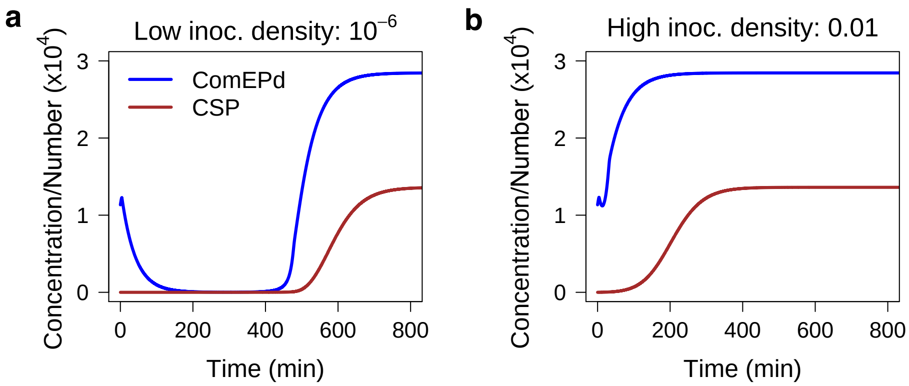

Supplementary Figure 3: Model predictions of the effect of inoculation density on competence development for cells that start being competent (mimicking preculture in non-acid conditions). The number of ComE$\sim$P dimer molecules per cell and the extracellular concentration of CSP over time are shown for two different inoculation densities. a) Initial density of 10^{-6}. b) Initial density of 0.01. Although cells are competent initially, if they are inoculated at low densities they can not produce enough CSP to remain competent and they switch off. On the other hand, if the inoculation density is high, cells produce enough CSP to remain competent. This explains the differences in the timing of competence initiation observed in Figures 2b and 2c. While for low inoculation densities there is no difference between the time of competence initiation for cells coming from acid and non-acid preculture, for high inoculation densities competence starts earlier for cells coming from non-acid preculture.

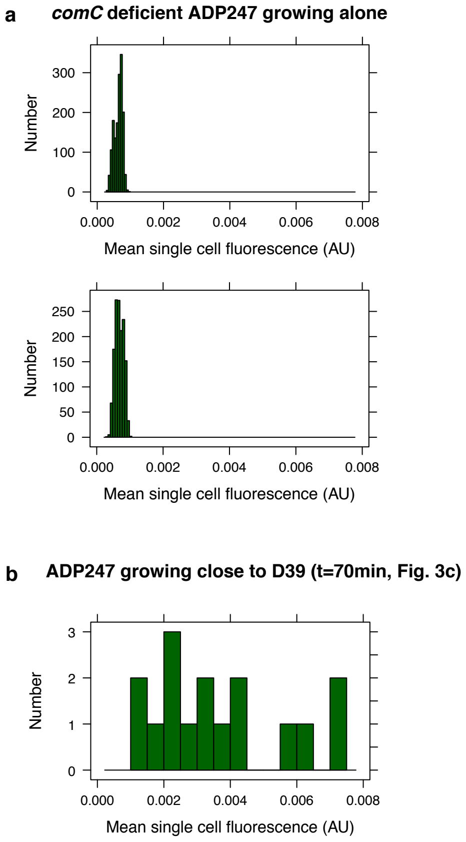

Supplementary Figure 4: The *comC* deficient ADP247 becomes competent only when grown with D39
a) Distributions of mean single-cell fluorescence signal in two different positions (top and bottom panel) on a slide with C+Y (pH 7.9) inoculated with only ADP247. The distribution includes every cell segmented in the first 110 minutes after inoculation to maximise the chance of detecting fluorescence signal if any. See Supplementary Movies 3 and 4 for the corresponding time-lapse videos with an overlay of phase contrast, GFP and RFP signal for the top and bottom panels respectively. b) Distribution of mean single-cell fluorescence signal at 70 minutes for the ADP247 colony growing close to the D39 colony in Figure 3c. Both strains were spotted together on a slide with C+Y (pH 7.9).

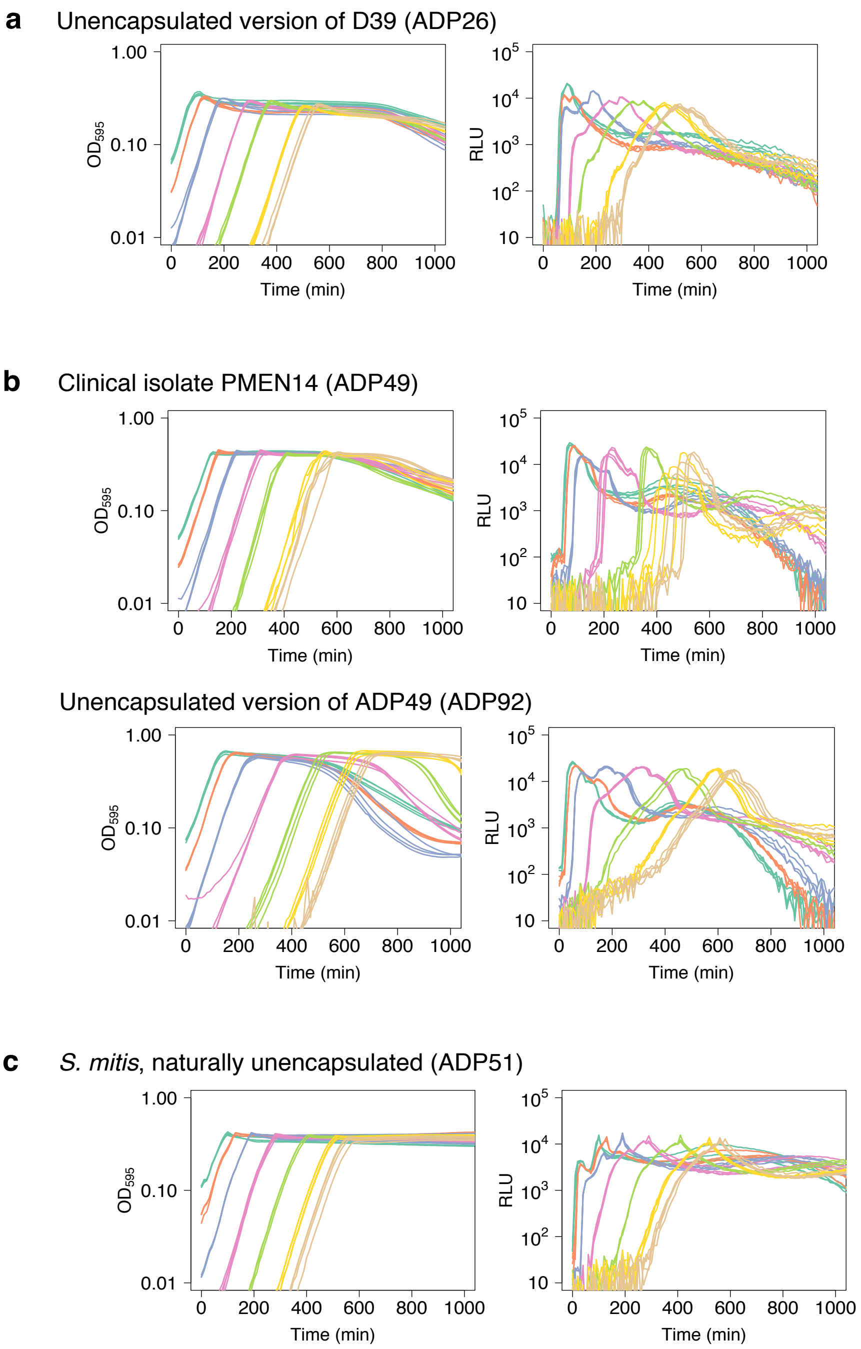

Supplementary Figure 5: Effect of cell density on competence development in other strains. Growth curves (OD_{595}) and competence expression measured as relative luminescence units (RLU) expressed from the promoter of the late competence gene *ssbB* for different strains. In all cases, cells were precultured in acid C+Y (pH 6.8) and inoculated at a range of densities in C+Y at pH 7.9. The inoculation densities were (OD_{595}): 0.1 (Green), 0.05 (Red), 0.01 (Blue), 10^{-3} (Purple), 10^{-4} (Green), 10^{-5} (Yellow) and 10^{-6} (Brown). Four replicates are shown per condition. a) ADP26, an unencapsulated version of DLA3 made by deleting the entire *cps* operon. b) ADP49, the clinical isolate PMEN14 with the *ssbB-luc* reporter, and its unencapsulated version ADP92. The entire *cps* operon was deleted from ADP49 to construct ADP92. c) ADP51, *S. mitis* with the ssbB-luc reporter. *S. mitis* is naturally unencapsulated. Although the time of competence initiation increases with lower inoculation density in all the strains, the slope of the RLU signal decreases with the inoculation density in the unecapsulated strains. This was also reported by Claverys *et al.*[9] and Prudhomme *et al.*[10] in their studies, which were made only with unencapsulated strains.

a

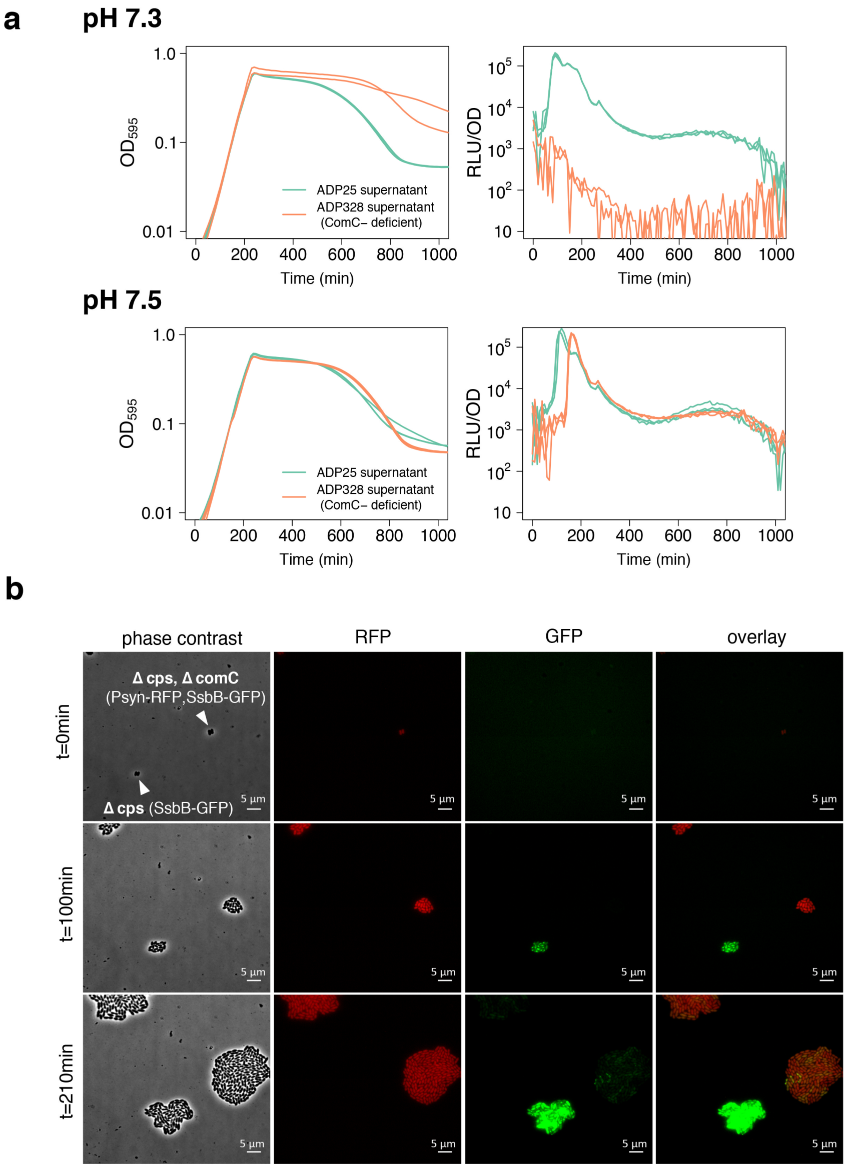

Supplementary Figure 6: The unencapsulated D39 releases CSP to the extracellular space and can spread competence without cell-cell contact. a) Growth curves and competence expression measured as relative luminescence units normalised by cell density (RLU/OD) for ADP26 cells growing in 2x concentrated C+Y mixed at a 1:1 ratio with cell-free supernatant from two cultures: one where competence developed, unencapsulated D39 (ADP25) grown at pH 7.9, and one where competence did not develop, ADP328 (*comC*-deficient ADP25) grown at pH 7.9. Three replicates are shown per condition. In a) the initial pH of the medium is adjusted to 7.3. Competence only develops when the supernatant from the competent culture was added to the medium. In b) the initial pH of the medium is adjusted to 7.5. Although at this pH competence develops both with the supernatant of the competent and non-competent cultures, it develops faster in the first case. This suggests that the supernatant from the competent unencapsulated D39 culture contained CSP that lead to competence induction of ADP26 at pH 7.3 and sped up competence induction at pH 7.5. b) Time-lapse fluorescence microscopy tracking competence development in two colonies with a fusion of the late competence gene *ssbB* to *gfp*: one formed by cells of the unencapsulated D39, ADP151, and one formed by cells of a *comC* deficient unencapsulated D39, ADP248. The two strains are distinguishable because ADP248 constitutively expresses a red fluorescent protein. Competence develops first in the unencapsulated D39 and then it propagates to the unencapsulated *comC* deficient mutant without the necessity of cell-cell contact. Scale bar: 5 μm.

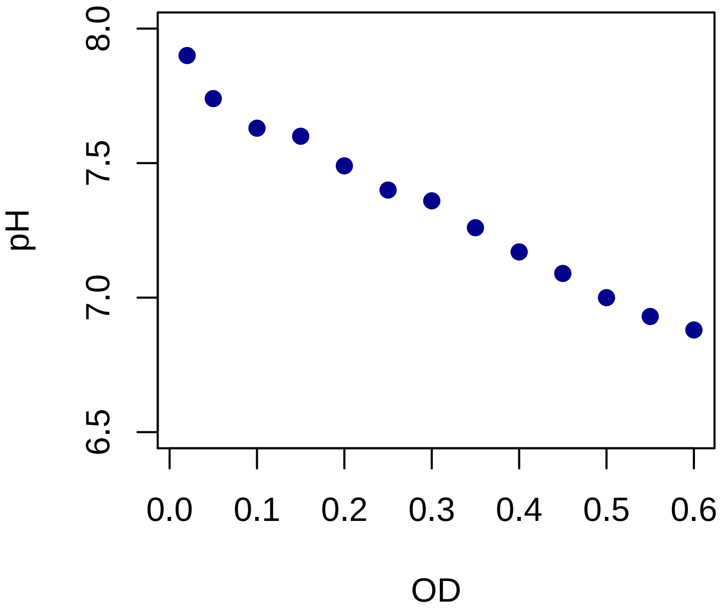

Supplementary Figure 7: pH decreases with cell density. Change of pH of the medium during DSM2 growth. The initial pH of the medium is 7.9. Although pH drops considerably during growth, by the density competence develops under our experimental conditions (below 0.1, see Figure 2c), acidification is still minor.

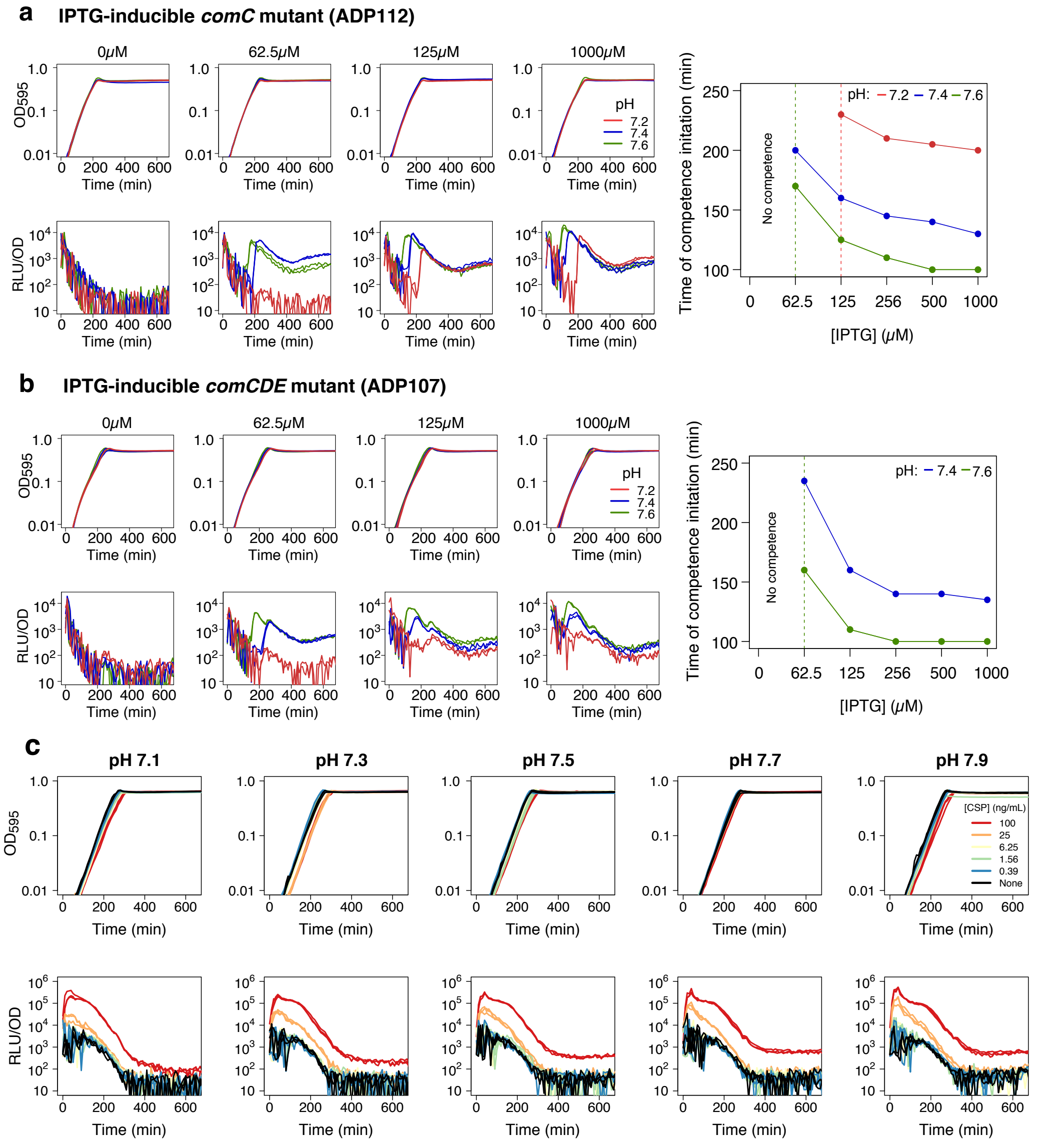

Supplementary Figure 8: Interplay of pH and external CSP in competence development. a) Growth curves and competence expression as RLU units normalized by cell density for the IPTG-inducible *comC* mutant, ADP112, at different IPTG concentration and pH values. For a fixed IPTG concentration competence develops faster with increasing pH. When the IPTG concentration is 62.5 μM competence does not even develop at pH 7.2. b) Growth curves and competence expression as RLU units normalized by cell density for the IPTG-inducible *comCDE* mutant, ADP107, at different IPTG concentrations and pH values. For a fixed IPTG concentration competence develops faster with increasing pH as observed for ADP112. The intensity of the RLU/OD signal decreases with pH and at pH 7.2 competence barely develops (the maximum total RLU signal is 350 or less for all the IPTG concentrations). Note that the ComE$\sim$P/ComE ratio in this mutant can be strongly lowered if cells export/detect less CSP with decreasing pH since ComE expression does not depend on this ratio (as in the wild type) but is instead fixed. This would explain reduced and almost absent competence development with decreasing pH in this mutant. IPTG is added at the moment of inoculation and two replicates are shown per condition in a) and b). The dots in the right graphs correspond to the average of the two. c) Growth curves and competence expression as RLU units normalized by cell density for populations of the *comC* deficient mutant ADP62 growing in C+Y medium with different pH and different initial concentrations of CSP. The colors represent the concentration of CSP that was externally added at the start of the culture: 100 ng mL^{-1} (Red), 25 ng mL^{-1} (Orange), 6.25 ng mL^{-1} (Yellow), 1.46 ng mL $^{-1}$ (Green), 0.39 ng mL^{-1} (Blue) and none (Black). Three replicates are shown for every condition.

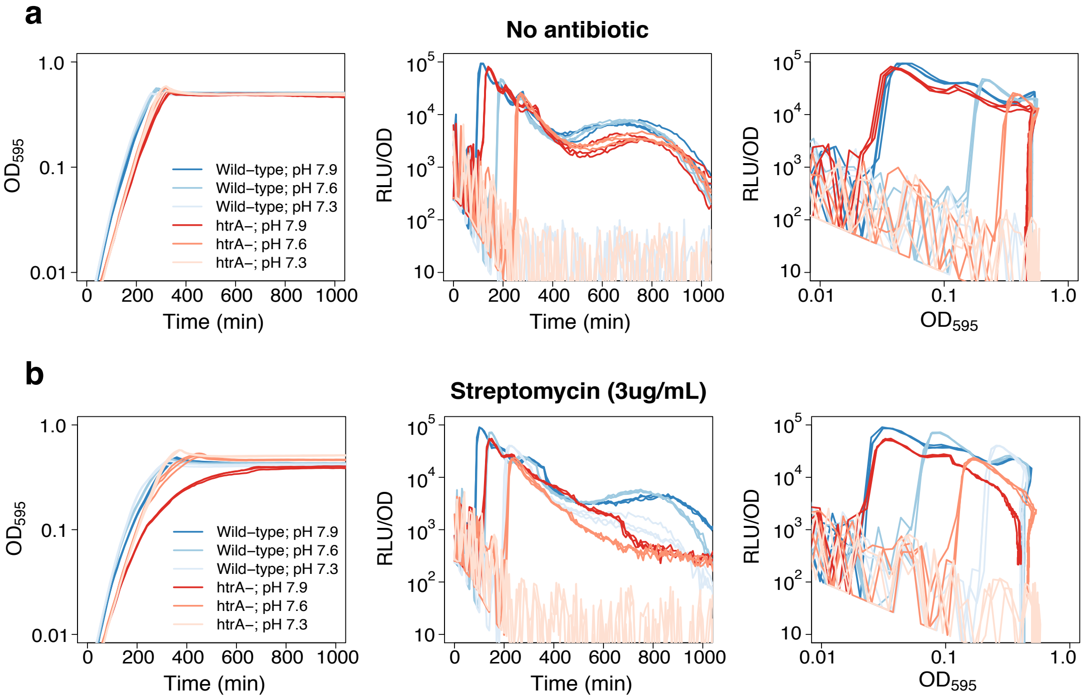

Supplementary Figure 9: Competence is not upregulated in an *htrA*$^-$ mutant relative to the wild type. Growth curves and competence expression at different pH (7.3, 7.6 and 7.9) for the wild type (in different shades of blue) and the *htrA*$^-$ mutant (in different shades of red) in a) medium with no antibiotic and b) medium with 3μg mL^{-1} of streptomycin. In a) competence does not develop in neither of the two strains at pH 7.3. In b) competence does develop in the wild type at pH 7.3 but not in the *htrA*$^-$ mutant. Based on the hypothesis that HtrA degrades CSP, it would be expected that competence is upregulated in the *htrA*$^-$ mutant relative to the wild type. We do not find support for this prediction since for the same pH competence always developed earlier in the wild type than in the *htrA*$^-$ mutant both in the presence and in the absence of streptomycin. Since the *htrA*$^-$ mutant grows at a lower rate, we also compared the density of competence initiation for the two strains (right graphs). With the exception of pH 7.9 with no antibiotic, competence always started at the same or at a higher density in the wild type than in the *htrA*$^-$ mutant.

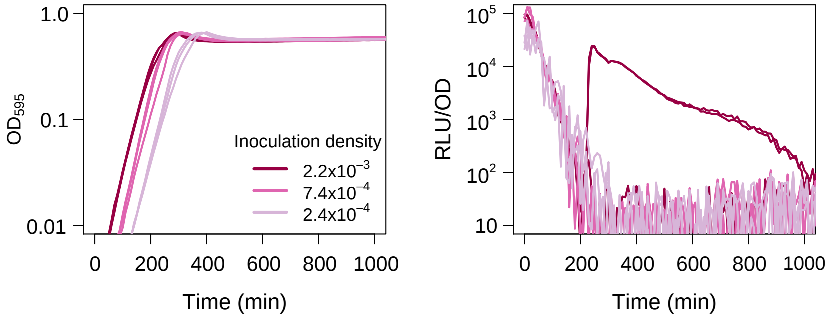

Supplementary Figure 10: Non-acid cell history can allow competence development also at pH 7.3 but a higher inoculation density is needed than at pH 7.4. Growth curves and competence expression as RLU units normalized by cell density (RLU/OD) for cells coming from non-acid preculture (pH 7.9) and inoculated in medium at pH 7.3 at different initial densities. Three replicates are shown per inoculation density. Competence only develops for the highest inoculation density as opposed to pH 7.4 where it also develops at OD_{595} 7.4×10^{-4}.

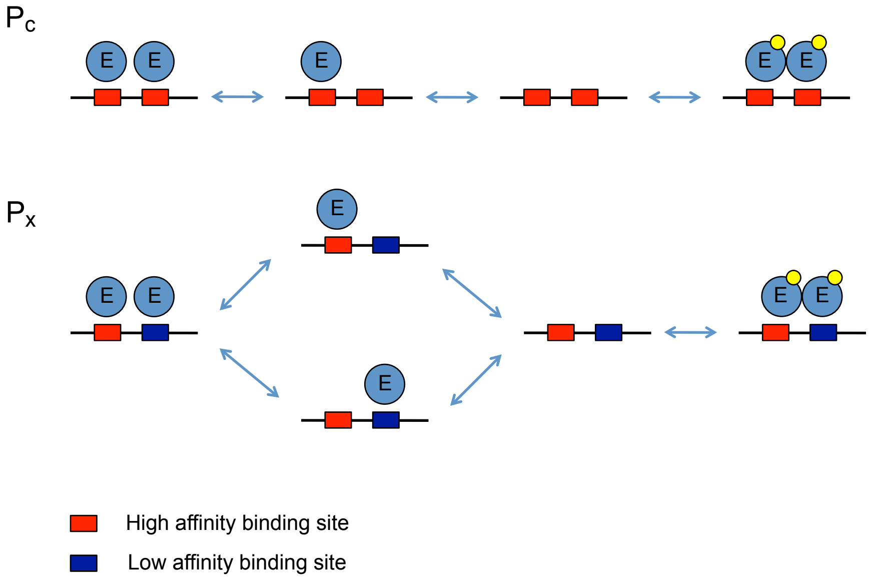

Supplementary Figure 11: Binding of ComE and ComE∼P to the two binding sites of the gene promoters P_c and P_c. Scheme based on the model suggested by Martin *et al.*[2] illustrating the possible configurations of the promoters. The yellow circles represent the phosphate groups of ComE∼P. We assume that transcription only happens at a high rate when the promoters are bound to the ComE∼P dimer. In all other configurations, transcription occurs at a basal rate.

Supplementary references

[1] D. Karlsson, S. Karlsson, E. Gustafsson, B.H. Normark, and P. Nilsson. Modeling the regulation of the competence-evoking quorum sensing network in *Streptococcus pneumoniae*. *Biosystems*, 90:211--23, 2007.

[2] B. Martin, A.L. Soulet, N. Mirouze, M. Prudhomme, I. Mortier-Barriére, C. Granadel, M.F. Noirot-Gros, P. Noirot, P. Polard, and J.P. Claverys. ComE/ComE$\sim$P interplay dictates activation or extinction status of pneumococcal X-state (competence). *Mol. Microbiol.*, 87:394--411, 2013.

[3] J. Slager, M. Kjos, L. Attaiech, and J.W. Veening. Antibiotic-induced replication stress triggers bacterial competence by increasing gene dosage near the origin. *Cell*, 157:395--406, 2014.

[4] K.E. Stevens, D. Chang, E.E. Zwack, and M.E. Sebert. Competence in *Streptococcus pneumoniae* is regulated by the rate of ribosomal decoding errors. *mBio*, 2:e00071--11, 2011.

[5] O.T. Avery, C.M. Macleod, and M. McCarty. Studies on the chemical nature of the substance inducing transformation of pneumococcal types. induction of transformation by a desoxyribonucleic acid fraction isolated from pneumococcus type iii. *J. Exp. Med.*, 79:137--158, 1944.

[6] S. Liang, M. Bipatnath, Y. Xu, S. Chen, P. Dennis, M. Ehrenberg, and H. Bremer. Activities of constitutive promoters in *Escherichia coli*. *J. Mol. Biol.*, 292:19--37, 1986.

[7] H.E. Kubitschek and J.A. Friske. Determination of bacterial cell volume with the coulter counter. *J. Bacteriol.*, 168:1466--1467, 1986.

[8] N.E. Buchler, U. Gerland, and T. Hwa. Nonlinear protein degradation and the function of genetic circuits. *Proc. Natl. Acad. Sci. U. S. A.*, 102:9559--9564, 2005.

[9] J.-P. Claverys, M. Prudhomme, and B. Martin. Induction of competence regulons as a general response to stress in gram-positive bacteria. *Annu. Rev. Microbiol.*, 60:451--475, 2006.

[10] M. Prudhomme, M. Berge, B. Martin, and P. Polard. Pneumococcal competence coordination relies on a cell-contact sensing mechanism. *PLoS Genet.*, 12:e1006113, 2016.
