# Supplementary material for: Quorum sensing integrates environmental cues, cell density and cell history to control bacterial competence
Source: Nat Commun. 2017 Oct 11;8:854. doi: 10.1038/s41467-017-00903-y (PMC5636887; doi:10.1038/s41467-017-00903-y)
Supplement: Supplementary file 3 — Description of Additional Supplementary Files [file 41467_2017_903_MOESM3_ESM.pdf]

Description of Additional Supplementary Files

File Name: Supplementary Movie 1
Description: Competence development synchronizes in microcolonies that are not in direct physical contact with each other. Time-lapse fluorescence microscopy tracking competence development of a set of neighboring microcolonies with a fusion of the late competence gene ssbB to gfp (same as in Figs. 3A and 3B). Competence starts after 60min and initiates in all microcolonies within a window of 5 minutes. Importantly, when competence initiates, the microcolonies are not in direct physical contact with each other. Scale bar: 5µm.

File Name: Supplementary Movie 2
Description: Competence spreads without the necessity of cell-cell contact. Time-lapse fluorescence microscopy tracking competence development in two colonies with a fusion of the late competence gene ssbB to gfp: one formed by cells of D39 (ADP249) and one formed by cells of a comC deficient D39 (ADP247). Overlay between phase contrast, GFP and RFP signals is shown. The two strains are distinguishable because ADP247 constitutively expresses a red fluorescent protein. Competence develops first in D39 and then it propagates to the comC- mutant without the necessity of cell-cell contact. Scale bar: 5µm.

File Name: Supplementary Movie 3
Description: Competence does not develop in the comC deficient D39 (ADP247) when grown alone. Time-lapse fluorescence microscopy tracking competence development in the comC- D39 (ADP247) grown in a slide where it has been inoculated alone (without the D39 strain). Overlay between phase contrast, GFP and RFP signals is shown. As expected, competence does not develop in this strain when growing alone since it does not produce any CSP. Scale bar: 5µm.

File Name: Supplementary Movie 4
Description: Replicate example similar to supplementary Movie S3 demonstrating that competence does not develop in the comC deficient D39 (ADP247) when grown alone. Time-lapse fluorescence microscopy tracking competence development in the comC- D39 (ADP247) grown in a slide where it has been inoculated alone (without the D39 strain). Overlay between phase contrast, GFP and RFP signals is shown. As expected, competence does not develop in this strain when growing alone since it does not produce any CSP. Scale bar: 5µm.

File Name: Supplementary Movie 5
Description: Competence spreads without the necessity of cell-cell contact also in unencapsulated pneumococci. Time-lapse fluorescence microscopy tracking competence development in two colonies with a fusion of the late competence gene ssbB to gfp: one formed by cells of an unencapsulated D39 (ADP151) and one formed by cells of a comC deficient unencapsulated D39 (ADP248). Overlay between phase contrast, GFP and RFP signals is shown. The two strains are distinguishable because ADP248 constitutively expresses a red fluorescent protein. Competence develops in the unencapsulated D39 and then it propagates to the comC- mutant without the necessity of cell-cell contact as observed for the encapsulated pneumococci. Scale bar: 5µm.
